# Studies of hepatitis E virus genotypes

**Published:** 2010-11

**Authors:** Yoon-Jae Song

**Affiliations:** Department of Life Science, Kyungwon University Seongnam-Si, Kyeonggi-Do, 461-701, Korea songyj@kyungwon.ac.kr

Hepatitis E virus (HEV) is the major aetiological agent of acute viral hepatitis and a member of the Hepeviridae family^1^. It has a single-stranded, positive RNA genome of 7.3 kb in length and contains 5’ untranslated region (UTR), three open reading frames (ORF 1, 2 and 3) encoding a non-structural protein, a capsid protein and a non structural phosphoprotein, respectively and 3’ UTR. Since there is no efficient cell culture system for HEV, detailed mechanisms of virus life cycle and pathogenesis are unclear.

A molecular phylogenetic analysis classifies HEV into four major genotypes^2^. Genotype 1 is found in developing countries in Asia and Africa, genotype 2 is isolated in Mexico and Africa, genotype 3 is distributed worldwide including developed countries, and genotype 4 is reported in Asia^2^. Genotype 3 and 4 are further divided into 10 (3a-3j) and 7 (4a-4g) subgenotypes, respectively, and found in both human and swine. Although the severity of HEV-associated acute hepatitis is believed to rely on the status of the host’s immune system, viral factors may also play important roles in the pathogenesis of the disease. Indeed, genotype of HEV contributes to the pathogenesis of HEV-associated hepatitis^3^. Genotype 4 HEV infected patients showed more severe form of the viral hepatitis than genotype 3 HEV infected patients^2^. Thus, the genetic changes in HEV genotypes may affect the effectiveness of virus transmission and, in turn, the severity of HEV-associated hepatitis. To further determine the transmission and pathogenesis of HEV, molecular epidemiological study of HEV genotypes are needed.

HEV is spread via the faecal-oral route and transmitted through water or raw food contaminated with faeces^1^. HEV is highly prevalent in developing countries with poor sanitation and hygiene. HEV endemic areas include central and south East Asia, northern and sub-Saharan Africa, the Middle East and Mexico^2^. In developed countries, sporadic HEV-associated hepatitis was diagnosed in person with a history of travel to HEV endemic regions. However, several cases of HEV-associated hepatitis were reported in developed countries among people who had no history of travel to endemic areas^4^–^7^. Although the cause of these incidents still needs to be determined, the zoonotic transmission of HEV, especially genotypes 3 and 4, was proposed because non-human primates, swine, sheep, cows, goats and rodents may serve as reservoirs for HEV^8^. Swine is considered to be a major reservoir of HEV infection because human HEV can experimentally infect swine and HEV isolates from human are genetically related to those from swine in the same geographic area^8^–^11^. However, India has been an exception to this hypothesis because genotype 1 HEV is mainly circulating in human and genotype 4 HEV in swine in this region^12^,^13^.

In this issue, Begum *et al*^14^ report a study investigating HEV genotype circulating in swine population from north India. As previously reported in other regions of India, genotype 4e HEV is predominant in swine from the region although the sample size test in their study is relatively small (67 samples). Interestingly, these HEV isolates from swine are genetically related to human isolates of India from 71.6 to 74.6 per cent indicating that a zoonosis may be a mode of transmission for HEV also in India. Indeed, a case of zoonotic transmission of HEV genotype 4 was reported in a patient with severe hepatitis and a history of travel to India^15^. This report further strengthens the hypothesis that the zoonosis is the mode of transmission for HEV. More molecular phylogenic analysis of HEV genotypes circulating in human and swine population in India is thus required to delineate the mode of HEV transmission and the evolution of new emerging HEV genotype subgroups.
